# Induction of osteoblastic differentiation of neural crest-derived stem cells from hair follicles

**DOI:** 10.1371/journal.pone.0174940

**Published:** 2017-04-06

**Authors:** Eri Urano-Morisawa, Masamichi Takami, Tetsuo Suzawa, Akifumi Matsumoto, Noriko Osumi, Kazuyoshi Baba, Ryutaro Kamijo

**Affiliations:** 1 Department of Biochemistry, School of Dentistry, Showa University, Tokyo, Japan; 2 Department of Prosthodontics, School of Dentistry, Showa University, Tokyo, Japan; 3 Department of Pharmacology, School of Dentistry, Showa University, Tokyo, Japan; 4 Division of Developmental Neuroscience, Tohoku University Graduate School of Medicine, Sendai, Japan; Universite de Nantes, FRANCE

## Abstract

The neural crest (NC) arises near the neural tube during embryo development. NC cells migrate throughout the embryo and have potential to differentiate into multiple cell types, such as peripheral nerves, glial, cardiac smooth muscle, endocrine, and pigment cells, and craniofacial bone. In the present study, we induced osteoblast-like cells using whisker follicles obtained from the NC of mice. Hair follicle cells derived from the NC labeled with enhanced green fluorescent protein (EGFP) were collected from protein zero-Cre/floxed-EGFP double transgenic mice and cultured, then treated and cultured in stem cell growth medium. After growth for 14 days, results of flow cytometry analysis showed that 95% of the EGFP-positive (EGFP^+^) hair follicle cells derived from the NC had proliferated and 76.2% of those expressed mesenchymal stem cells markers, such as platelet-derived growth factor α and stem cell antigen-1, and also showed constitutive expression of Runx2 mRNA. Cells stimulated with bone morphogenetic protein-2 expressed osteocalcin, osterix, and alkaline phosphatase mRNA, resulting in production of mineralized matrices, which were detected by von Kossa and alizarin red staining. Moreover, EGFP^+^ hair follicle cells consistently expressed macrophage colony-stimulating factor and osteoprotegerin (OPG). Addition of 1α,25-dihydroxyvitamin D_3_ [1,25(OH)_2_D_3_] (10^−8^ M) to the cultures suppressed OPG expression and induced RANKL production in the cells. Furthermore, multinucleated osteoclasts appeared within 6 days after starting co-cultures of bone marrow cells with EGFP^+^ cells in the presence of 1,25(OH)_2_D_3_ and PGE_2_. These results suggest that NC-derived hair follicle cells possess a capacity for osteoblastic differentiation and may be useful for developing new bone regenerative medicine therapies.

## Introduction

Neural crest cells (NCCs), a specific population of vertebrate cells originating in the dorsal neural tube [1, 2], form a variety of tissues, including the dorsal root ganglia, peripheral nerves, pigment and adipose cells, and craniofacial bone and muscle tissues [3–6]. In addition, certain cells in hair follicles appear to be derived from the neural crest (NC) [7–9]. Thus, NCCs are considered to possess multipotential characteristics and show significant migratory ability for distribution throughout the body.

Recent studies have indicated that undifferentiated cells are present in adult NC-derived tissues and organs, and that neural crest-derived cells (NCDCs) possess partial stem-cell properties, such as self-renewal and differentiation [8, 10–12]. Various transgenic mice have been developed to analyze the distribution and functions of NCDCs [13–17], with NC-specific Cre recombinase applied for genetic marking of NCDCs in mice, such as the protein zero (P0)-Cre and Wnt1-Cre strains [13, 14]. Kanakubo et al. [16] crossed P0-Cre Tg with CAG-CAT-EGFP Tg mice [18] to establish a transgenic line in which NCCs were genetically marked with enhanced green fluorescent protein (EGFP), and these P0-Cre/Floxed-EGFP double transgenic (P0-Cre; CAG-CAT-EGFP Tg) mice have been widely used to study NCDCs [19–23]. In one of those previous studies, NCDCs were identified and isolated from bone marrow, dorsal root ganglia, and whisker follicles obtained from adult P0-Cre; CAG-CAT-EGFP Tg mice [20]. In another, multipotent NCDCs from the iris stroma of those mice showed great potential as a cell source for regenerative treatment of damaged corneal tissues [19].

Osteoblasts play a central role in bone formation. Although osteoblast precursor cells are derived from the mesoderm, NCDCs also differentiate into osteoblasts in some cranial facial bone tissues, such as mandibular bone [5, 24–26], and several studies have also reported the differentiation of NCCs into osteoblast-like cells [17]. The process of differentiation of these cells is controlled by cell-specific expression of transcription factors, including Runx2 and osterix. Osteoblasts express different bone matrix proteins during the various stages of differentiation, e.g., pre-osteoblasts express alkaline phosphatase (ALP) and type 1 collagen, while mature osteoblasts express osteocalcin [27]. In addition, osteoblasts form matrix vesicles, which contain various enzymes and physiologically active substances, such as ALP and osteocalcin, and initiate early calcification [28], with calcified hard tissues often detected using alizarin red and von Kossa staining [29, 30].

In addition to producing bone matrix, osteoblasts also support differentiation of osteoclasts via the activity of receptor activator of nuclear factor-κB ligand (RANKL), a cytokine known to mediate osteoclast differentiation [31]. Osteoblasts also produce macrophage colony-stimulating factor (M-CSF), which stimulates osteoclast progenitor cells, resulting in increased proliferation and differentiation. Various factors such as 1α,25-dihydroxyvitamin D_3_ [1,25(OH)_2_D_3_] and prostaglandin E_2_ (PGE_2_) stimulate osteoblasts to express RANKL on the surface of their membranes subsequent to stimulation [32]. Furthermore, osteoblasts suppress osteoclast differentiation via expression of osteoprotegerin (OPG), which serves as a decoy receptor of RANKL [33, 34].

Studies of bone grafting have been conducted using autogenous, allogeneic, and artificial bone tissues [35, 36]. To regenerate functional bone tissue using tissue engineering, 5 characteristics are required; osteoconductive and osteoinductive properties, osteogenic ability, immune rejection-free status, and mechanical load-bearing ability [36–39]. Autogenous bone combines all of those properties, although the limited availability of that for bone grafts and surgical stress in patients restricts its use [40, 41].

In order to reduce invasive bone regeneration using stem cells, hair follicles, which can be removed with a low level of surgical stress, can be utilized. Those are known to contain stem cells [42–44], with the dermal papilla (DP) in particular reported to retain stem cell-like properties and the hair follicle bulge area (bulge) to contain adult stem cells [42, 43]. In addition, hair follicle stem cells have been shown to have potential for osteoblast differentiation [45–47], although detailed findings indicating their differentiation potential have not been presented.

In the present study, to induce osteoblasts from NC-derived hair follicle cells (NCDFCs), we obtained NCDFCs labeled with EGFP from P0-Cre; CAG-CAT-EGFP Tg mice and cultured them in the presence of BMP-2. Our results demonstrated that NCDFCs express osteoblast differentiation markers and produce mineralized matrix in response to BMP-2. Additionally, we confirmed that NCDFCs support osteoclast differentiation, suggesting their capacity for osteoblast differentiation. These properties would be useful for developing new methods for bone tissue engineering.

## Materials and methods

### Ethics statement

All experimental procedures utilized in this study were approved by the Ethical Board for Animal Experiments of Showa University (Approval No. 14048). All mice were observed daily, with cage cleaning and feeding performed once a week. Euthanasia was conducted by cervical dislocation in a manner to alleviate distress and suffering according to the protocol outlined in the Animal Experiment Manual of Showa University.

### Animals

Transgenic mice expressing Cre recombinase driven by the myelin protein zero (P0) promoter [13] were mated with CAG-CAT-EGFP transgenic mice [18]. NCDCs in the P0-Cre; CAG-CAT-EGFP Tg mice were identified by evaluating GFP expression subsequent to P0-Cre-mediated DNA recombination [16]. To confirm the genotype, polymerase chain reaction (PCR) analyses for P0-Cre and CAG-CAT-EGFP were performed as previously described [13, 18].

### Preparation of hair follicle cells

To culture hair follicle cells, whisker hair follicles were removed from 8- to 16-week-old P0 mice, following euthanasia by cervical dislocation. Approximately 50 hair follicles were obtained from 2 different mice for use in each of the experiments. After peeling face skin, follicles were removed with tweezers (#3 Peer-Vigor) under a stereomicroscope, then washed in phosphate-buffered saline, and digested with 0.1% collagenase (Wako Pure Chemical Industries, Ltd., Osaka, Japan) and 0.2% Dispase II (Wako Pure Chemical Industries) in Dulbecco's Modified Eagle Medium (DMEM)-F12 (Wako Pure Chemical Industries) for 1 hour at 37°C. To evaluate the proliferative potential of hair follicle cells, dissociated cells were cultured in stem cell growth medium composed of DMEM-F12 (1:1) supplemented with 20 ng/mL recombinant human epidermal growth factor (Invitrogen Co., Carlsbad, USA), 20 ng/mL human fibroblastic growth factor-basic (Invitrogen), and B27 supplement (Life Technologies, Carlsbad, CA, USA) in 6-cm dishes coated with collagen gel (Cell Matrix Type 1-A collagen; Nitta Gelatin Inc., Osaka, Japan). Next, cells were passaged in 10-cm dishes coated with collagen gel and allowed to grow to confluence, then treated with 0.1% collagenase and 0.2% Dispase II in Dulbecco's Modified Eagle Medium (DMEM)-F12 for 5–10 minutes to dissolve the collagen gel. Finally, cells were collected by centrifugation and allowed to passage in 10-cm collagen Type I-coated dishes (IWAKI, AGC Techno Glass Co, Ltd., Shizuoka, Japan).

### Induction of osteoblastic differentiation

Proliferative hair follicle cells (5×10^5^ cells/dish) were cultured in α Minimum Essential Medium (αMEM; Wako Pure Chemical Industries) containing 10% fetal calf serum (FCS) and 200 ng/mL recombinant human BMP-2 (R&D Systems, Inc., MN, USA) in 6-cm collagen Type I-coated dishes (IWAKI). Fresh medium was added on day 3 of the culturing process. Osteoblastic differentiation was determined by measuring ALP activity, ALP staining, and expression of osteoblast-related genes (Runx2, ALP, osteocalcin, osterix). To confirm calcification, proliferative hair follicle cells (2×10^4^ cells/well) were cultured in αMEM containing 10% FCS, 200 ng/mL recombinant human BMP-2, 10 mM β-glycerophosphate (Sigma-Aldrich, St. Louis, MO, USA), 50 mg/mL ascorbic acid (Sigma-Aldrich), and 10^−8^ M dexamethasone (Sigma-Aldrich) in 96-well collagen Type I-coated plates (IWAKI) for 20 days. Calcification was detected by von Kossa and alizarin red staining.

### Induction of osteoclastic differentiation

Following euthanasia performed by cervical dislocation, bone marrow cells (approximately 2×10^6^ cells/ml) were obtained from the tibiae and femora of 4- to 6-week-old male ddY strain mice (Sankyo Labo Service Corporation, Inc., Tokyo, Japan) using a syringe equipped with a 21-G needle (Terumo, Inc., Tokyo, Japan), and used in the experiments. Proliferative hair follicle (1×10^4^ cells/well) and bone marrow (1×10^5^ cells/well) cells were co-cultured in αMEM containing 10% FCS, 10^−8^ M 1,25(OH)_2_D_3_, and 10^−6^ M PGE_2_ for 6 days in 96-well adherent cell culture plates (Thermo Fisher Scientific, Waltham, MA, USA). To detect osteoclast formation, cells were fixed and stained with tartrate-resistant acid phosphatase (TRAP), which is an osteoclast marker. Fast red violet LB salt (Sigma-Aldrich) and naphthol AS-MX phosphate (Sigma-Aldrich) were dissolved in 0.1 M acetic buffer (pH 5.0) containing 1% tartaric acid [48]. The cytoskeleton was detected by staining cultured cells with fluorescein isothiocyanate (FITC)-phalloidin (Life Technologies), for which cells were first rinsed in phosphate-buffered saline containing 0.2% Triton X-100 (Nacalai Tesque, Inc., Kyoto, Japan) and then incubated with 1 μM FITC- phalloidin for 1 day at 4°C. For pit formation assays, proliferative hair follicle (1×10^6^ cells/dish) and bone marrow (1×10^7^ cells/dish) cells were co-cultured in αMEM containing 10% FCS, 10^−8^ M 1,25(OH)_2_D_3_, and 10^−6^ M PGE_2_ for 6 days in 10-cm dishes coated with collagen gel. Next, proliferating cells were placed on dentin slices (0.3 mm thick, 6 mm in diameter) in αMEM containing 10% FCS, 10^−8^ M 1,25(OH)_2_D_3_, and 10^−6^ M PGE_2_ for 6 days in 96-well adherent cell culture plates. After culturing for 31 hours, to visualize resorption pits, cells were removed from the dentin slices with a cotton swab and stained with toluidine blue O (Sigma-Aldrich). In addition, osteoclastic differentiation was determined by measuring expression levels of osteoclast-related genes, such as nuclear factor of activated T-cells cytoplasmic1 (NFATc1), osteoclast-associated receptor (OSCAR), and calcitonin receptor. To determine the expressions of RANKL, OPG, and M-CSF on osteoblast membranes after stimulation with RANKL expression-stimulating factors such as 1,25(OH)_2_D_3_, we determined the mRNA expression levels of RANKL, OPG, and M-CSF.

### Reverse transcription-PCR (RT-PCR)

Total RNA of cultured cells was extracted using TRIzol reagent (Invitrogen). First-strand cDNA was synthesized using Superscript III (Life Technologies) and subjected to amplification with GoTaq DNA polymerase (Promega, Madison, WI, USA), with the following specific PCR primer pairs: GAPDH, 5′-AACTTTGGCATTGTGGAAGG-3′ (forward) and 5′-CCCTGTTGCTGTAGCCGTAT-3′ (reverse); p75, 5′-CATCTCTCTGTGGACAGCCAGA-3′ (forward) and 5′-TCTGTGGGGGCTAGAACATC-3′ (reverse); Twist1, 5′-CGGACAAGCTGAGCAAGATT-3′ (forward) and 5′-GGGACACAAACGAGTGTGTTCA-3′ (reverse); Snail1, 5′-GAGGACAGTGGCAAAAGCTC-3′ (forward) and 5′-CTTCACATCCGAGTGGGTTT-3′ (reverse); ALP, 5′-AACCCAGACACAAGCATTCC-3′ (forward) and 5′-CTGGGCCTGGTAGTTGTTGT-3′ (reverse); Runx2, 5′-CAGACCAGCAGCACTCCATA-3′ (forward) and 5′-CTGCCTCTTGTCCCTTTCTG-3′ (reverse); osteocalcin, 5′-CAGACAAGTCCCACACAGCA-3′ (forward) and 5′-ACTTGCAGGGCAGAGAGAGA-3′ (reverse); osterix, 5′-TGCTTCCCAATCCTATTTGC-3′ (forward) and 5′-AGAATCCCTTTCCCTCTCCA-3′ (reverse); NFATc1, 5′-TCATCCTGTCCAACACCAAA-3′ (forward) and 5′-TTGCGGAAAGGTGGTATCTC-3′ (reverse); OSCAR (osteoclast-associated receptor), 5′-ACTCCTGGGATCAACGTGAC-3′ (forward) and 5′-GATAGCACATAGGGGGCAGA-3′ (reverse); CTR, 5′-CTGCTCCTAGTGAGCCCAAC-3′ (forward) and 5′-CAGCAATCGACAAGGAGTGA-3′ (reverse); M-CSF, 5′-GCTCCTGCCTACCAAGACTG-3′ (forward) and 5′-TATGCCTTTACGGGAAGTCG-3′ (reverse); RANKL, 5′-AGCCGAGACTACGGCAAGTA-3′ (forward) and 5′-GATGGTGAGGTGTGCAAATG-3′ (reverse); and OPG, 5′-GAACCCCAGAGCGAAATACA-3′ (forward) and 5′-CGCTGTTTTCACAGAGGTCA-3′ (reverse). The PCR assay protocol was 30 cycles at 94°C, 57°C, and 72°C for 10, 20, and 30 seconds, respectively.

### Flow cytometry analysis

FACSVerse^TM^ (BD Biosciences, Franklin Lakes, NJ, USA) was utilized to identify the level of proliferative EGFP-positive (EGFP^+^) cells as well as that of mesenchymal stem cells among proliferative EGFP^+^ cells. Both freshly isolated and proliferative hair follicle cells were stained with anti-platelet-derived growth factor α (PDGFRα) and anti-stem cell antigen-1 (Sca-1) (e-Bioscience, Santa Clara, CA, USA) for 30 minutes on ice, and then compared.

### Histological analysis

For histological analysis, whisker hair follicles were removed from 8- to 16-week-old P0 mice, following euthanasia by cervical dislocation, then fixed in 4% paraformaldehyde at 4°C overnight and additionally washed with 30% sucrose in PBS at 4°C overnight. Next, the samples were embedded in O.C.T. compound (Sakura Finetek Japan Co., Ltd., Tokyo, Japan) and cut using a cryostat (Leica Microsystems K.K., Tokyo, Japan) as preparation for HE staining and immunohistochemistry. For immunohistochemistry, frozen sections were washed with 0.1% Triton X-100 (Nacalai Tesque) in PBS (PBST), and blocked with 3% BSA (Sigma-Aldrich) and 1% sheep serum (Sigma-Aldrich) in PBST for 1 hour at 37°C. The sections were incubated with anti-GFP (#A-11122, rabbit IgG, 1:1000, Invitrogen) as the primary antibody overnight at 4°C, then rinsed 3 times with PBST and Alexa Fluor^®^ 594 (#A-11012, goat anti-rabbit IgG, 1:500, Invitrogen) as the secondary antibody for 1 hour at 37°C. As a negative control, 3% BSA and 1% sheep serum in PBST were used instead of the primary antibody. Sections were observed under a BIOREVO BZ-9000 confocal microscope (Keyence, Osaka, Japan).

### Statistical analysis

Student’s t-test was used for statistical analysis, with p values less than 0.05 considered to be significant.

## Results

### Distribution of neural crest-derived cells in whisker follicles

**Fig 1:**
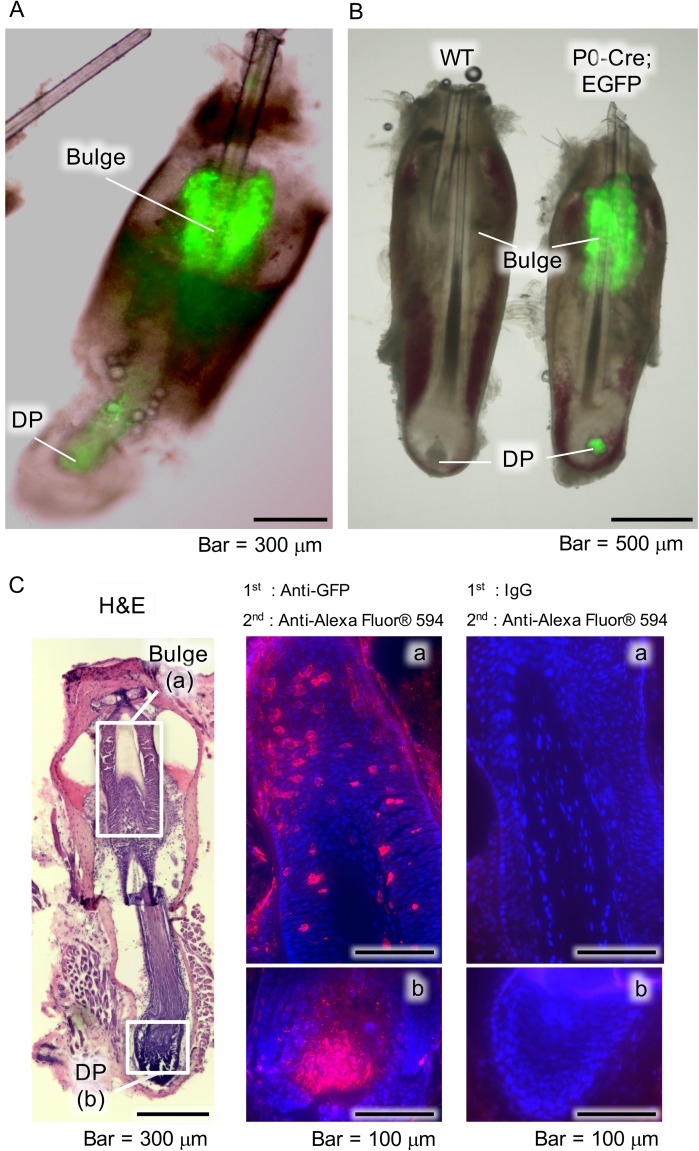
Distribution of NCDCs in whisker follicles. (A) Fluorescence microscopy images of whisker follicle samples obtained from P0-Cre; CAG-CAT-EGFP Tg mice. Bulge, hair follicle bulge area; DP, dermal papilla. (B) Fluorescence microscopy images of whisker follicle samples obtained from wild-type (WT) and P0-Cre; CAG-CAT-EGFP Tg (P0-Cre; EGFP) mice. (C) Histological and immunohistochemical analysis findings of whisker follicles from P0-Cre; CAG-CAT-EGFP Tg mice following hematoxylin and eosin staining (left panel), and immunostaining with anti-GFP (middle panel), as well as of the negative control (right panel). Boxed areas in left panel indicate the bulge (a) and DP (b), which are shown at higher magnification in the middle and right panels.

The distribution of EGFP-tagged NCDCs in whisker follicle samples obtained from P0-Cre; CAG-CAT-EGFP Tg mice was determined using fluorescence microscopy. EGFP^+^ cells were observed in the bulge (Fig 1A and 1B) and those findings were confirmed by immunohistochemistry. In the sections, we demonstrated EGFP^+^ cells were red fluorescent cells using an Alexa Fluor^®^ 594 (red fluorescent antibody) secondary antibody (bulge, Fig 1C-a; DP, Fig 1 C-b). In the negative control samples, no red fluorescent cells were detected in the bulge or DP (Fig 1C-right panel). These results showed the existence of NCDCs in the bulge and DP.

### NCDFCs proliferated in stem cell growth medium and expressed mesenchymal stem cell markers

**Fig 2:**
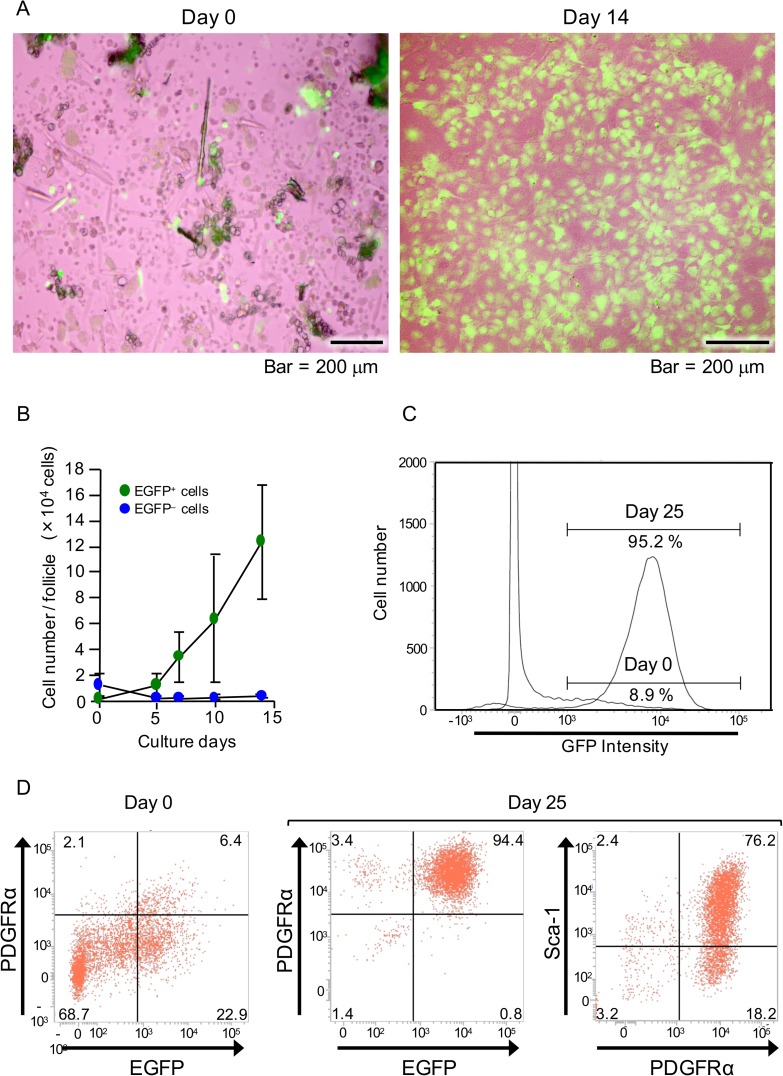
Examination of proliferative potential and level of mesenchymal stem cells in NCDFCs. (A) Phase-contrast images of whisker follicle cells subsequent to enzymatic processing on day 0 (left panel) and cells cultured in stem cell growth medium for 14 days (right panel). (B) The number of EGFP^+^ cells per whisker follicle increased with culture time. (C) EGFP-gated flow cytometry analysis charts of whisker follicle cells. EGFP^+^ cells were detected immediately after sampling (day 0) as well as after 25 days of proliferation in stem cell growth medium. (D) Flow cytometry analysis of cell-surface markers using freshly isolated whisker follicle cells from P0-Cre; CAG-CAT-EGFP Tg mice (day 0) and after 25 days of proliferation in stem cell growth medium. Representative flow cytometry images revealing EGFP- and PDGFRα-gated (left and middle panels) cells, and PDGFRα- and Sca-1-gated cells (right panel). Data shown represent mean values from 3 independent experiments, with error bars indicating SD.

Next, the proliferative potential of whisker follicle cells was examined. Subsequent to enzymatic processing of the bulge and DP, EGFP^+^ cells were cultured and found to grow well in stem cell growth medium up to 14 days (Fig 2A). In addition, as shown in images obtained with phase-contrast and fluorescence imaging, green fluorescence indicated EGFP cells (S1 Fig). Flow cytometry analysis showed that the number of EGFP^+^ cells increased each day (Fig 2B), eventually reaching greater than 95% of total cells (Fig 2C), while their proliferation speed was also significantly greater than that of the EGFP^-^ cells. Additionally, we identified mesenchymal stem cell markers in the NCDFCs using flow cytometric analysis. EGFP^+^ cells expressed the mesenchymal stem cell marker PDGFRα and proliferated in stem cell growth medium, and 76.2% of the cells expressed the mesenchymal stem cell marker Sca-1 (Fig 2D). Thus, approximately 4.3×10^3^ cells obtained from 50 hair follicles of 2 mice were shown to be mesenchymal stem cells derived from the neural crest. These results suggest that NCDFCs have high proliferative potential when cultured in stem cell growth medium, with many of the proliferating cells found to be identical to PDGFRα^+^ and Sca-1^+^ mesenchymal stem cells.

### NCDFCs differentiated into osteoblasts following BMP-2 stimulation

**Fig 3:**
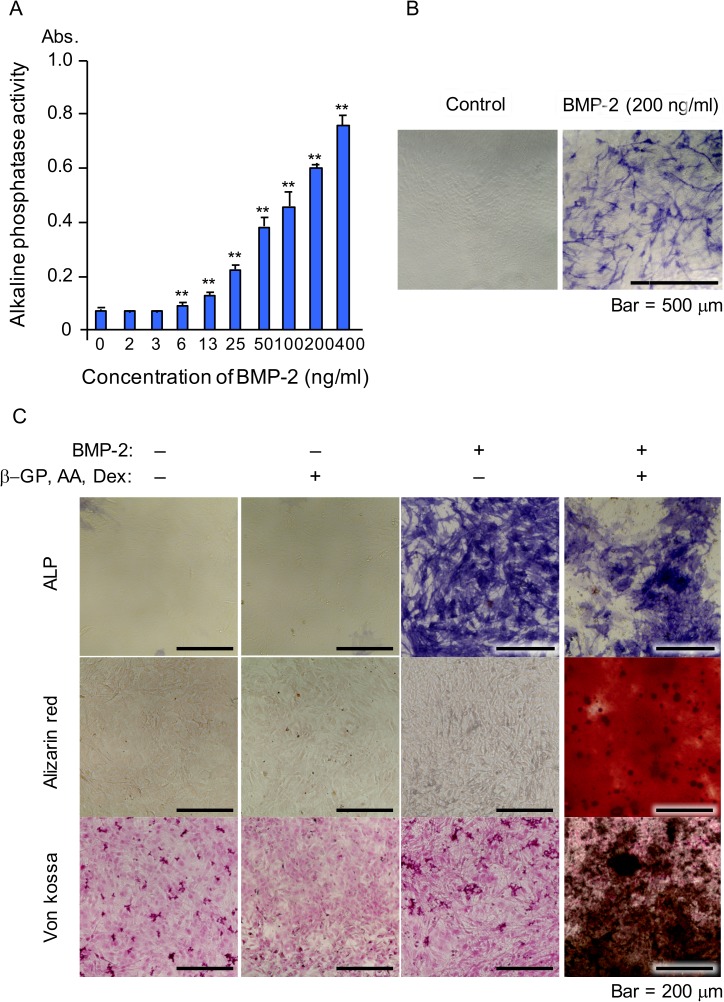
Osteogenic differentiation of NCDFCs. (A, B) Analysis of ALP expression. (A) ALP activity was elevated following addition of BMP-2 in a dose-dependent manner; Abs, absorbance. (B) Positive staining for ALP following addition of BMP-2. (C) Detection of calcification in calcification medium following alizarin red (middle panel), and von Kossa (lower panel) staining, and ALP expression (upper panel). β-GP, β-glycerophosphate; AA, ascorbic acid; Dex, dexamethasone. Data shown represent mean values from 3 independent experiments, with error bars indicating SD. **P<0.01.

We also analyzed the potential of NCDFCs to differentiate into osteoblasts in response to BMP-2, an inducer of osteoblast differentiation. Proliferative EGFP^+^ cells cultured in the presence of BMP-2 for 5 days expressed ALP in a BMP-2 dose-dependent manner (Fig 3A and 3B). In addition, calcification was detected by alizarin red and von Kossa staining after 20 days of culture in the presence of ascorbic acid, β-glycerophosphate, dexamethasone, and BMP-2 (Fig 3C). Ours results suggested that NCDFCs differentiated into osteoblasts following stimulation with BMP-2.

### BMP-2 induced expression of osteoblast-related genes in NCDFCs

**Fig 4:**
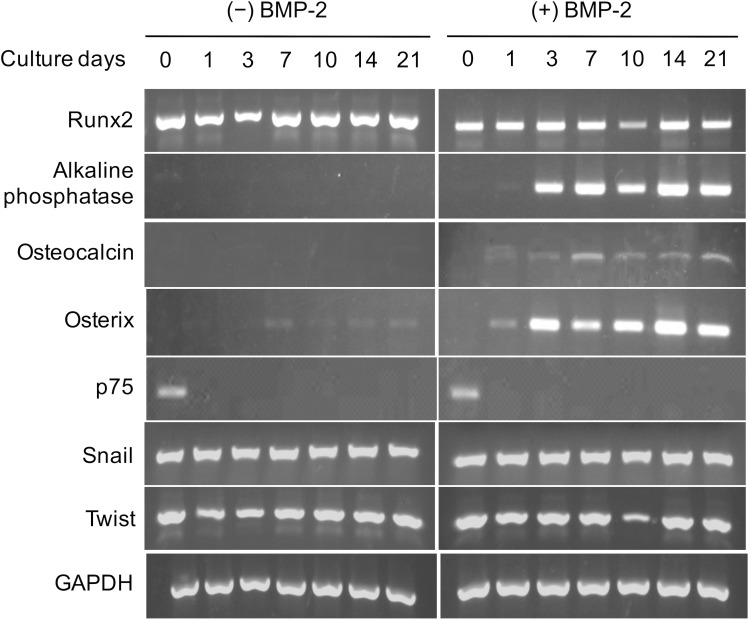
Expression patterns of osteoblast-related and NC cell-related genes in NCDFCs. Proliferative NCDFCs were stimulated with or without BMP-2, then mRNA expression levels were analyzed over time. All cells continuously expressed Runx2 mRNA, while stimulation with BMP-2 induced expressions of the osteoblastic differentiation-related genes ALP, osterix, and osteocalcin. In contrast, expression of p75, an NC cell-related gene, was not seen with or without BMP-2.

Proliferative EGFP^+^ cells were stimulated with or without BMP-2, then mRNA expression levels were measured (Fig 4). The cells were found to express Runx2 mRNA prior to BMP-2 stimulation, while the expressions of ALP, osterix, and osteocalcin, each of which are osteoblastic differentiation-related genes, were observed following stimulation. In contrast, the expression of the NC-related gene p75 was suppressed with or without BMP-2, while expression of the NC-related genes Snail and Twist did not vary over time (Fig 4). These results also support the notion that that NCDFCs possess an ability to differentiate into osteoblasts.

### NCDFCs supported osteoclast differentiation

**Fig 5:**
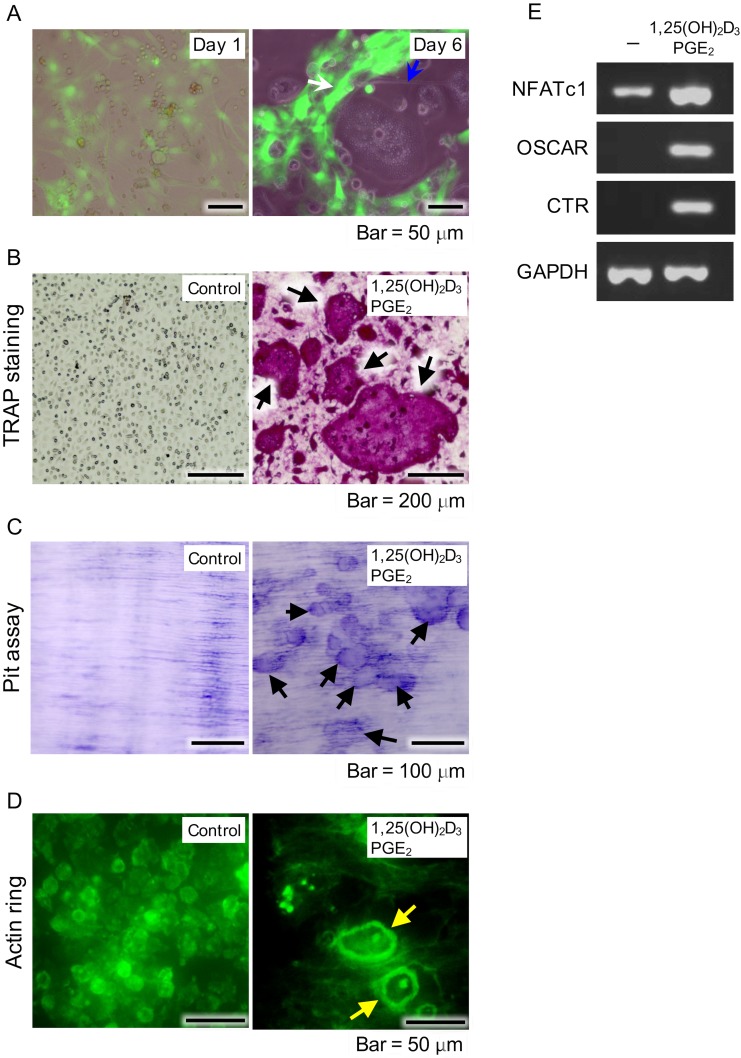
Examination of osteoblast functions supporting differentiation of osteoclasts. (A) NCDFCs co-cultured with mouse bone-marrow cells including osteoclast precursor cells. Phase-contrast images of proliferative NCDFCs and mouse bone marrow cells in the presence of PGE_2_ and 1,25(OH)_2_D_3_ on day 1 (left panel). Formation of osteoclast-like cells (blue arrow in right panel) alongside EGFP^+^ cells (white arrow in right panel) on day 6. (B) Formation of osteoclasts in presence of PGE_2_ and 1,25(OH)_2_D_3_ detected by TRAP staining (arrows) after 6 days. (C) Cells showed bone resorption capacity after 6 days, as detected by toluidine blue staining (arrows). (D) Cells formed actin rings specific to the cytoskeleton of osteoclasts (yellow arrows), as detected by FITC-phalloidin staining after 6 days. (E) Semi quantitative RT-PCR measurements of expression of the osteoclast-related genes NFATc1, OSCAR, and calcitonin receptor in the presence of 1,25(OH)_2_D_3_ and PGE_2_. PGE_2_, prostaglandin E_2_; RT-PCR, reverse transcription-polymerase chain reaction.

In addition to their role in bone matrix production, osteoblasts also support osteoclast differentiation through RANKL production. To examine whether EGFP^+^ cells support osteoclast differentiation, we co-cultured proliferative EGFP^+^ and mouse bone marrow cells in the presence of PGE_2_ and 1,25(OH)_2_D_3_. Addition of 1,25(OH)_2_D_3_ and PGE_2_ to the cultures resulted in formation of osteoclast-like giant cells (Fig 5A). After culturing for 6 days, those cells were confirmed to be osteoclasts, as shown by TRAP staining (Fig 5B) and bone resorption capacity assay findings (Fig 5C), while evidence of formation of a specific cytoskeletal structure known as actin rings was also observed (Fig 5D). In addition, these cells expressed the osteoclast-related genes NFATc1, OSCAR, and calcitonin receptor after 6 days of culture (Fig 5E), suggesting that osteoblasts and NCDFCs support osteoclast differentiation. As for the control experiments, TRAP staining detected no osteoclasts in cultures of bone marrow cells or NCDFCs alone (S2 Fig).

### NCDFCs supported osteoclast differentiation via RANKL production

**Fig 6:**
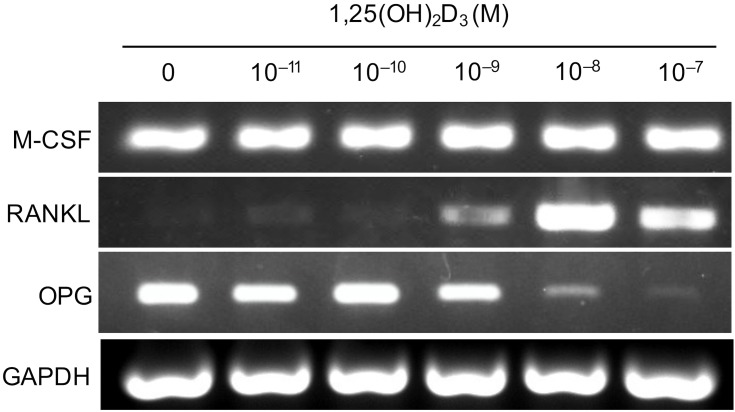
Analysis of mRNA expression levels in proliferative NCDFCs stimulated with various concentrations of 1,25(OH)_2_D_3_. RT-PCR analysis of NCDFCs exposed to osteoclast differentiation stimulating factors (M-CSF, RANKL) and an osteoclast differentiation inhibiting factor (OPG). Proliferative NCDFCs continuously expressed M-CSF. Addition of 1,25(OH)_2_D_3_ to the cultures suppressed the expression of OPG and induced production of RANKL by the cells.

To determine whether RANKL is involved in osteoclast differentiation, we stimulated proliferative EGFP^+^ cells with various concentrations of 1,25(OH)_2_D_3_ and analyzed mRNA expression levels. EGFP^+^ cells produced M-CSF and OPG prior to stimulation, whereas M-CSF expression by the cells did not appear to be modulated by 1,25(OH)_2_D_3_. Addition of 1,25(OH)_2_D_3_ to the cultures suppressed the expression of OPG and induced production of RANKL by the cells (Fig 6). These results indicate that NCDFCs support osteoclast differentiation via production of RANKL.

## Discussion

In the present study, we were able to efficiently obtain an increased number of NCDFCs, as representative EGFP^+^ cells grew well until finally comprising greater than 95% of the total cell population after 14 days. In addition, we identified the presence of EGFP^+^ cells in bulge and DP specimens. Previous studies have demonstrated the existence of NC stem cells in the DP of adult mouse hair follicles, while it has also been reported that pluripotent NC stem cells were found to be contained within adult mouse whisker follicles residing specifically in the outer-root sheath of the bulge to matrix at the base of the follicle [7, 8]. Furthermore, the presence of NCDCs in the DP of whisker follicles has been reported [20]. The present results support those previous findings, and additionally suggest that NCDCs in the bulge and DP possess stem cell characteristics, and have an ability to differentiate into osteoblasts.

NCCs appear during the formation of the neural tube, during which they emigrate from epithelium into the mesoderm layer, creating mesenchymal tissues (epithelial to mesenchymal transition; EMT). After undergoing EMT, NCCs migrate to various locations in the embryo and differentiate into a diverse variety cells at the destination [49, 50]. In addition, Oshima et al. reported that the bulge contains stem and/or progenitor cells whose migration is indispensable for proper whisker growth [43]. Kellner et al. also noted that the bulge contains adult stem cells, while cells in the DP retain their stem cell-like properties [42]. Our results demonstrated that proliferative EGFP^+^ cells express the mesenchymal stem cell markers Sca-1 and PDGFRα [51], indicating possession of stem cell characteristics. Interestingly, we found that EGFP^+^ cells proliferated well in stem cell growth medium, while EGFP^−^ cells did not show similar growth, indicating that the former and not the latter possess stem cell characteristics. Furthermore, our findings suggest that such a culture environment is best suited for EGFP^+^ cells. We concluded that the present culturing method using stem cell growth medium, collagen gel, and collagen Type I-coated dishes is appropriate and suitable for NCDFC growth.

In a previous study, Yu et al. isolated and expanded adult stem cells from human hair follicles by culturing in human embryonic stem cell medium [52], then in a later study they induced osteoblast differentiation and evaluated differentiation by ALP staining [53]. Jahoda et al. suggested that adult hair follicle DP and dermal sheath-derived clones were capable of osteoblast differentiation, which was evaluated by Von Kossa and osteopontin staining [45]. However, they did not analyze alterations in gene expression of osteoblast differentiation markers or examine BMP-2, a factor well known to have an essential role in osteoblast differentiation [54–56]. In the present experiments, we used 200 ng/ml BMP-2 for osteogenic differentiation of NCDFCs, because Katagiri et al. reported that a concentration of 300 ng/ml was a powerful regulator for determining osteoblast differentiation, as conversion of the differentiation pathway of C2C12 myoblasts into an osteoblast lineage was observed at that concentration, while BMP-2 at 100 ng/ml did not show such effects [57].

Our results clearly demonstrated that BMP-2 stimulates NCDFCs to differentiate into osteoblasts, which was confirmed by evidence of altered gene expression profiles. We also found that BMP-2 elevated ALP activity and induced expression of the osteoblastic differentiation-related genes ALP, osteocalcin, and osterix. In addition, we detected calcification by alizarin red and von Kossa staining in the presence of BMP-2, ascorbic acid, dexamethasone, and β-glycerophosphate, and also observed that Runx2, a transcription factor essential for osteoblastic differentiation [27, 58–60], was expressed in the present NCDFCs. Glotzer et al. reported that expression of Runx2 in hair follicles regulates their development and is involved in determining skin thickness during morphogenesis [61]. Since Runx2 is also essential for osteoblast differentiation, NCDFCs were shown to differentiate into osteoblasts.

Previous studies have reported that RANKL is induced in the plasma membrane of osteoblasts in response to bone-resorbing factors such as 1,25(OH)_2_D_3_, parathyroid hormone, and PGE_2_, and that interleukin-11 mediates essential signalling to osteoclast progenitor cells, leading to their differentiation into mature osteoclasts [31, 62]. Osteoclast progenitors possessing RANK recognize RANKL by cell-to-cell contact and differentiate into osteoclasts. Also, osteoblasts produce M-CSF, which is indispensable for both proliferation and differentiation of osteoclast progenitors, as well as OPG, a decoy receptor for RANKL [33]. Thus, osteoblasts are important for osteoclast differentiation [63]. In addition to osteoblasts, osteoblast precursor cells, such as bone marrow-derived stromal cells and the mouse marrow-derived stromal cell line ST2, have been reported to support osteoclast differentiation [64–66]. Interestingly, we observed osteoclast differentiation in the presence of proliferative EGFP^+^ cells regardless of stimulation by BMP-2 (S3 Fig), suggesting that support for the potential of osteoclast differentiation by NCDFCs is equivalent to that of osteoblasts and related precursors. In addition, stimulation of proliferative EGFP^+^ cells with various concentrations of 1,25(OH)_2_D_3_ induced RANKL expression and suppressed OPG in a 1,25(OH)_2_D_3_ dose-dependent manner, while M-CSF was continuously expressed in proliferative EGFP^+^ cells. Thus, osteoblasts as well as NCDFCs are able to control osteoclast differentiation.

Bone tissues have higher regeneration potential than cartilage, thus normal fractures and small bone defects are generally treated with conservative management [67–69]. However, complicated fractures and larger bone defects that do not naturally heal are treated with bone grafting generally consisting of autogenous, allogeneic, or artificial bone. Use of autogenous bone requires invasive surgery of healthy tissue and is clinically limited by supply, while allogeneic bone may be rejected by the immune system. Although use of artificial bone circumvents these issues and is not limited by supply, that constructed from materials such as hydroxyapatite and β-tricalcium phosphate is inorganic, and formation of new bone *in vivo* with exclusive use of artificial bone is difficult. Therefore, new types of bone grafts created with cultured autologous cells have recently been reported. In particular, bone marrow mesenchymal stem cells and adipose tissue have received attention, with successful bone tissue engineering with those cell types reported [70–73]. The present results demonstrated that NCDFCs possess the capability of osteoblastic differentiation following stimulation with BMP-2, indicating their potential use as a cell source for bone tissue engineering. Nevertheless, additional studies are needed to address their *in vivo* osteogenic capability.

## Supporting information

S1 Fig Phase-contrast and green fluorescence images of proliferative NCDFCs. (Left panel) Phase-contrast image of proliferative NCDFCs. (Right panel) Green fluorescence image of proliferative NCDFCs. Green fluorescence shown in ***Fig 2A*** is from EGFP cells.
(TIF)

S2 Fig Control experiments of proliferative NCDFCs treated without BMP-2 to support their differentiation into osteoclasts. Proliferative NC-derived hair follicle (1×10^4^ cells/well) and bone marrow (1×10^5^ cells/well) cells were co-cultured in αMEM containing 10% FCS, 10^−8^ M 1,25(OH)_2_D_3_, and 10^−6^ M PGE_2_ for 10 days in 96-well adherent cell culture plates. BM, bone marrow cells; NCDFCs, NC-derived hair follicle cells. To detect osteoclast formation, cells were fixed and stained with TRAP. Arrows indicate osteoclasts. (Upper panel) TRAP stained cells were not detected in cultures with only BM. (Middle panel) TRAP stained cells were not detected in cultures with only NCDFCs. (Lower panel) TRAP stained cells were detected in co-cultures of BMs and NCDFCs with 1,25(OH)_2_D_3_ and PGE_2_, without BMP-2.
(TIF)

S3 Fig Experiments using proliferative NCDFCs treated with BMP-2 for support of osteoclast differentiation. Proliferative NC-derived hair follicle (1×10^4^ cells/well) and bone marrow (1×10^5^ cells/well) cells were co-cultured in αMEM containing 10% FCS, 10^−8^ M 1,25(OH)_2_D_3_, and 10^−6^ M PGE_2_ in the presence of BMP-2 for 10 days in 96-well adherent cell culture plates. BM, bone marrow cells; NCDFCs, NC-derived hair follicle cells. To detect osteoclast formation, cells were fixed and stained with TRAP. Arrows indicate osteoclasts. (Upper panel) TRAP stained cells were not detected in cultures with only NCDFCs. (Lower panel) TRAP stained cells were detected in co-cultures of BM and NCDFCs with 1,25(OH)_2_D_3_ and PGE_2_ in the presence of BMP-2.
(TIF)
