# Supplementary figures and images for: Induction of osteoblastic differentiation of neural crest-derived stem cells from hair follicles
Source: PLoS One. 2017 Apr 6;12(4):e0174940. doi: 10.1371/journal.pone.0174940 (PMC5383073; doi:10.1371/journal.pone.0174940)

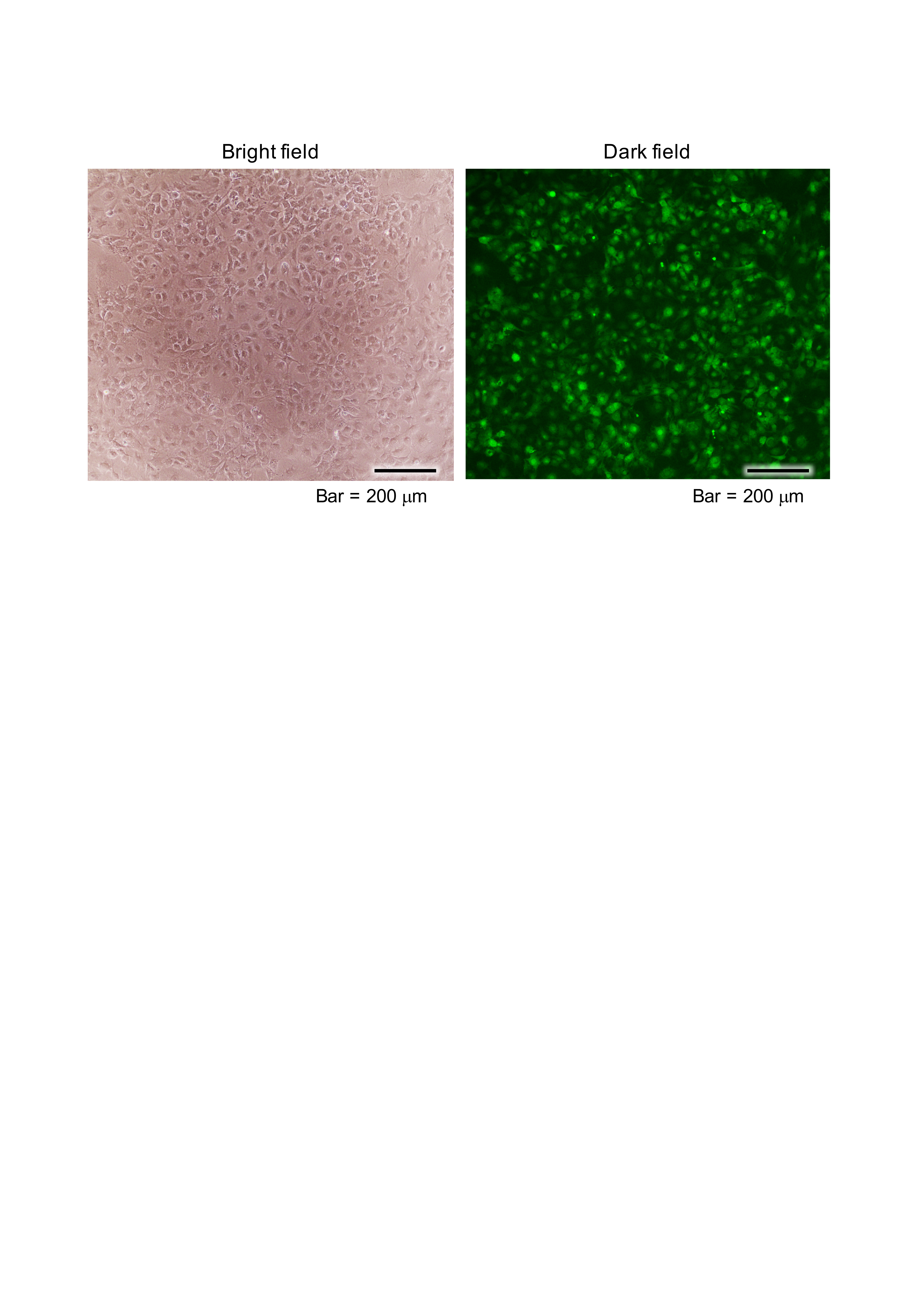

Supplement: S1 Fig — (Left panel) Phase-contrast image of proliferative NCDFCs. (Right panel) Green fluorescence image of proliferative NCDFCs. Green fluorescence shown in Fig 2A is from EGFP cells. (TIF) [file pone.0174940.s001.tif]

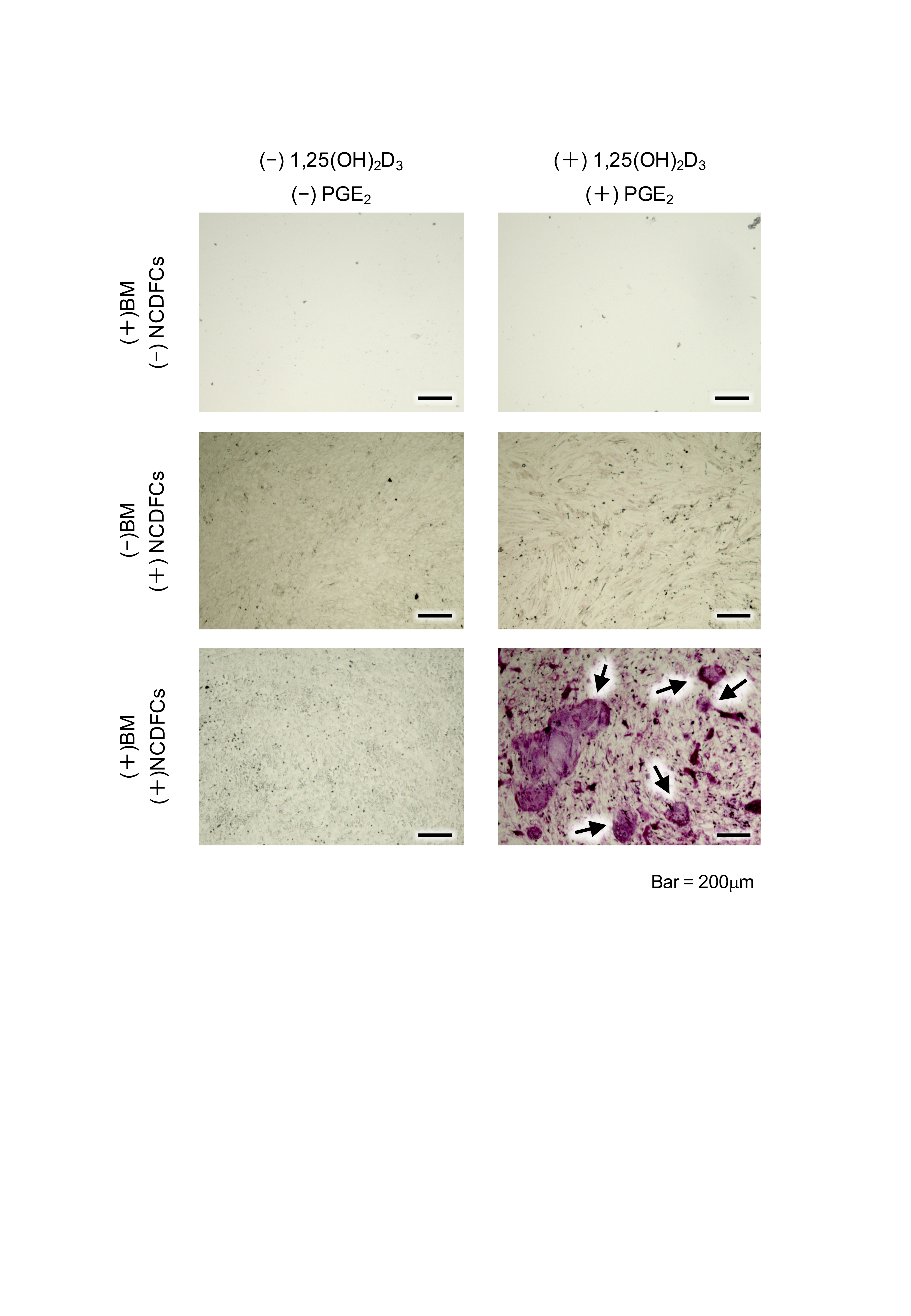

Supplement: S2 Fig — Proliferative NC-derived hair follicle (1×10^4^ cells/well) and bone marrow (1×10^5^ cells/well) cells were co-cultured in αMEM containing 10% FCS, 10−8 M 1,25(OH)2D3, and 10−6 M PGE2 for 10 days in 96-well adherent cell culture plates. BM, bone marrow cells; NCDFCs, NC-derived hair follicle cells. To detect osteoclast formation, cells were fixed and stained with TRAP. Arrows indicate osteoclasts. (Upper panel) TRAP stained cells were not detected in cultures with only BM. (Middle panel) TRAP stained cells were not detected in cultures with only NCDFCs. (Lower panel) TRAP stained cells were detected in co-cultures of BMs and NCDFCs with 1,25(OH)2D3 and PGE2, without BMP-2. (TIF) [file pone.0174940.s002.tif]

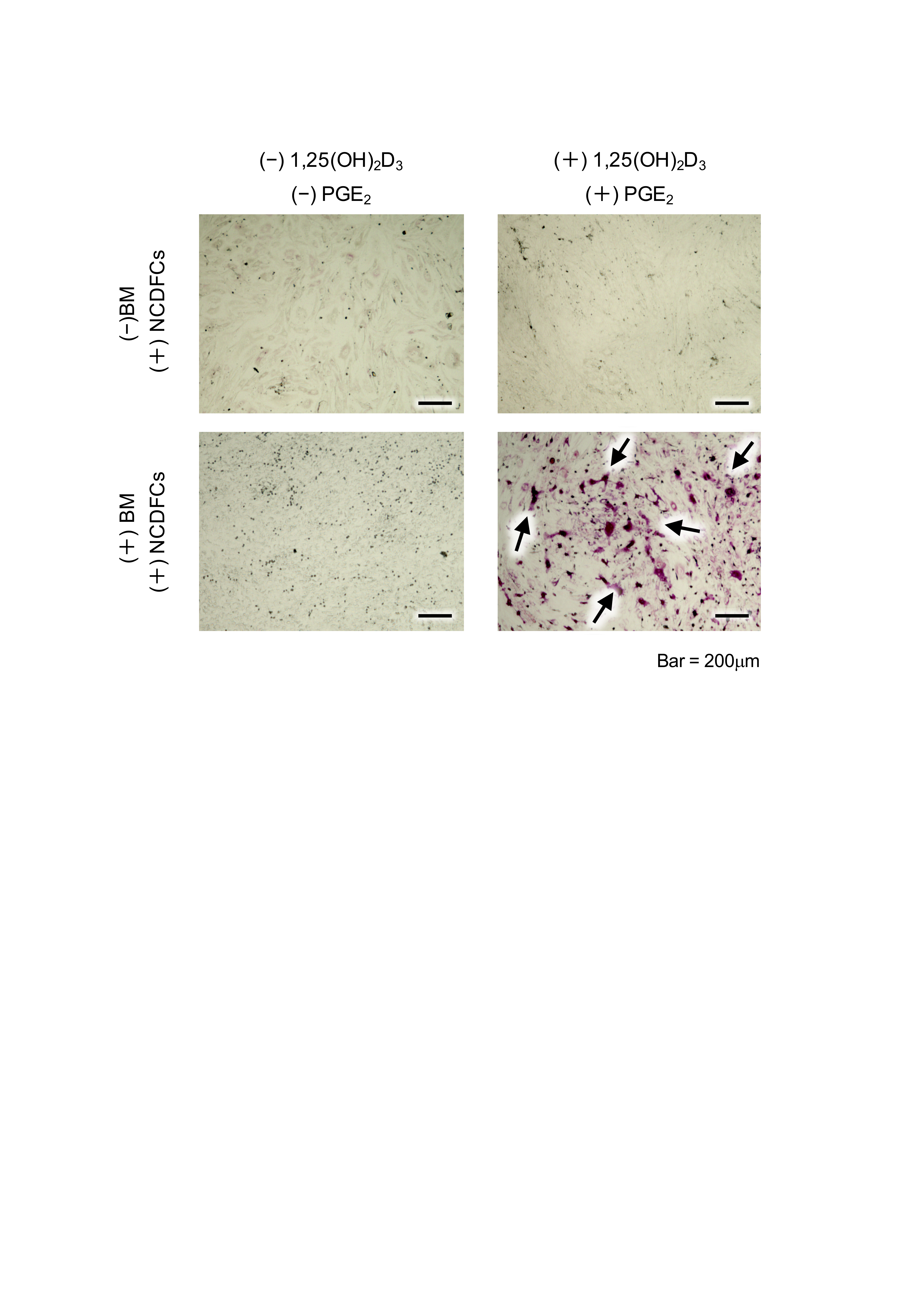

Supplement: S3 Fig — Proliferative NC-derived hair follicle (1×10^4^ cells/well) and bone marrow (1×10^5^ cells/well) cells were co-cultured in αMEM containing 10% FCS, 10−8 M 1,25(OH)2D3, and 10−6 M PGE2 in the presence of BMP-2 for 10 days in 96-well adherent cell culture plates. BM, bone marrow cells; NCDFCs, NC-derived hair follicle cells. To detect osteoclast formation, cells were fixed and stained with TRAP. Arrows indicate osteoclasts. (Upper panel) TRAP stained cells were not detected in cultures with only NCDFCs. (Lower panel) TRAP stained cells were detected in co-cultures of BM and NCDFCs with 1,25(OH)2D3 and PGE2 in the presence of BMP-2. (TIF) [file pone.0174940.s003.tif]
